# Clinical Outcomes After Immediate Coronary Angiography in Elderly Versus Younger Patients Suffering from Acute Coronary Syndrome

**DOI:** 10.3390/jcdd12090362

**Published:** 2025-09-17

**Authors:** Anja Radunovic, Ivan Ilic, Milica Matic, Miljana Ostojic, Dejan Kojic, Ana Golocevac, Nikola Lazarevic, Aleksandar Mandic, Milosav Tomovic, Petar Otasevic

**Affiliations:** 1 Department of Cardiology, Institute for Cardiovascular Diseases Dedinje, 11000 Belgrade, Serbia; anja.radunovic95@gmail.com (A.R.); ivan1ilic@yahoo.com (I.I.); milica.matic@ymail.com (M.M.); miljanaostojic99@gmail.com (M.O.); kojicdrd@yahoo.com (D.K.); anamatovic888@gmail.com (A.G.); lazarevich.nikola96@gmail.com (N.L.); aleksandar.mandic1993@gmail.com (A.M.); milosavtomovic@gmail.com (M.T.); 2 Faculty of Medicine, University of Belgrade, Dr Subotića Street 8, 11000 Belgrade, Serbia

**Keywords:** acute coronary syndrome, elderly, PCI, DAPT, P2Y12 inhibitors

## Abstract

(1) Aims: This study aimed to compare cardiovascular outcomes in patients older than 75 years with those of younger patients who underwent interventional treatment for acute coronary syndrome (ACS) at a tertiary university hospital. (2) Methods and Results: This was a retrospective, observational study conducted between January 2016 and December 2021, including 1846 consecutive patients with ACS (older than 75 years *n* = 203, 11%; younger than 75 years *n* = 1643, 89%). After admission, patients underwent coronary angiography and subsequently received percutaneous coronary intervention (PCI), coronary artery bypass grafting (CABG), or medical therapy. The mean age in the older group (O75) was 80 ± 4 years versus 59 ± 9 years in the younger group (Y75) (*p* < 0.001). Older patients more frequently presented with multivessel coronary disease (O75: 114 [56%] vs. Y75: 727 [44%], *p* = 0.004), and the left anterior descending artery (LAD) was more often the culprit vessel (O75: 105 [52%] vs. Y75: 684 [41%]). Major adverse cardio-cerebral events (MACCEs) occurred more frequently in patients older than 75 years, mainly due to higher mortality (O75: 14 [6.9%] vs. Y75: 27 [1.6%], *p* < 0.001) and stroke (O75: 3 [1.5%] vs. Y75: 2 [0.1%], *p* < 0.001). Multivessel disease was the only factor independently associated with MACCEs (HR 1.417, 95% CI 1.058–1.898, *p* = 0.02). The incidence of significant bleeding (Bleeding Association Research Consortium (BARC) class ≥ 3) was similar between groups (Y75: 123/1643 [7.5%] vs. O75: 13/203 [6.5%], *p* = 0.587). (3) Conclusions: Patients older than 75 years have worse short- and long-term prognoses following ACS compared with younger patients. Special attention and a multidisciplinary, personalized approach are required to optimize outcomes in this population.

## 1. Introduction

Increased life expectancy, driven by improved living conditions and advances in medical care, has led to a growing number of elderly patients presenting with more complex coronary artery disease (CAD). Consequently, the number of elderly patients with acute coronary syndrome (ACS) undergoing invasive diagnostics and treatment has steadily increased [1]. Despite the aging population, there is no universal consensus on the definition of “elderly.” However, in daily clinical practice, patients older than 75 years are generally considered elderly, in line with current European and American cardiology guidelines for the management of ACS [2,3].

In ACS patients, irrespective of whether they receive medical or interventional therapy, the foundation of treatment is dual antiplatelet therapy (DAPT), typically consisting of acetylsalicylic acid (ASA) and a potent P2Y12 receptor antagonist. Among patients undergoing percutaneous coronary intervention (PCI) with stent implantation, the primary goal of DAPT is to prevent stent thrombosis and recurrent atherothrombotic events. ACS itself, caused by atherosclerotic plaque rupture and the resulting prothrombotic state, carries an inherently increased risk of thrombosis.

Elderly patients frequently present with comorbidities that further amplify their risks following ACS and its treatment [4]. In fact, in clinical practice, nearly one-third of patients undergoing PCI are classified as being at high bleeding risk (HBR), largely due to factors such as advanced age, atrial fibrillation, prior stroke, chronic kidney disease, bleeding disorders, or the presence of prosthetic heart valves. These conditions significantly increase the risk of adverse cardiovascular events and the likelihood of clinically relevant bleeding when DAPT is prescribed [5].

Early interventional treatment in ACS patients has been advocated in order to reduce adverse cardiovascular events, especially in patients presenting with high-risk features [2,3]. However, there is a paucity of data regarding the potential benefits of early invasive strategies for elderly patients due to their aforementioned characteristics. Early invasive strategies could relieve myocardial ischemia, but they could compromise kidney function and cause vascular complications and bleeding, which can easily offset the benefits of improved hemodynamics.

Given these challenges, the aim of our study was to compare cardiovascular and bleeding outcomes in ACS patients older than 75 years with those of younger patients who underwent immediate interventional treatment in a tertiary-level hospital. In addition, we sought to examine the characteristics of interventions performed in the elderly compared with their younger counterparts.

## 2. Materials and Methods

A total of 1846 consecutive patients admitted with ACS [ST elevation myocardial infarction (STEMI), non-ST elevation myocardial infarction (NSTEMI), and unstable angina (UA)], were included in this retrospective observational study conducted at a university cardiovascular hospital from January 2016 to December 2021. They underwent immediate coronary angiography (within 6 h of admission) and received subsequent treatment with PCI, surgical revascularization, or no revascularization—medical therapy alone was included in the study. Antithrombotic regimen was DAPT that included ASA and P2Y12 receptor inhibitor (ticagrelor, prasugrel, or clopidogrel). After hospitalization, patients were discharged with a recommendation for 12-month DAPT. All of them were treated with a high dose of a potent statin, except in the case of known hypersensitivity or adverse reactions to the agent, when other lipid-lowering treatments were used. Other medications were given according to the current guidelines for ACS [2,3].

Patients were divided based on age cutoff, set at 75 years of age.

Inclusion criteria:

- Admission with ACS (STEMI, NSTEMI, or UA).
- Underwent coronary angiography within 6 h of admission.
- Age ≥ 18 years.

Exclusion criteria:

▪ No ACS diagnosis.
▪ Killip class IV at admission.
▪ Requirement for mechanical circulatory support (intra-aortic balloon pump, Impella micro-axial pump), and/or mechanical ventilation.
▪ Significant valvular disease or prior valve surgery.
▪ Major in-hospital bleeding requiring surgery or transfusion.
▪ Anticipated life expectancy < 1 year due to non-cardiac conditions.

Data collection was based on the institutional database, encompassing records of hospital admissions and outpatient visits for each patient and supplemented by telephone interviews conducted by study personnel. All collected data were systematically entered into a dedicated electronic database developed for the purposes of this study. Data collected in the registry were as follows: demographic data—unique identification number, age, sex, height, weight, phone, address; previous medical history—myocardial infarction (MI), cerebrovascular insults (CVIs), revascularization procedures including PCI or coronary artery bypass grafting (CABG); risk factors for atherosclerosis—heredity for cardiovascular diseases, smoking, diabetes mellitus (DM), hypertension (HTA), dyslipidemia, peripheral arterial disease (PAD); PCI data—number of coronary arteries with more than 50% stenosis, access site for PCI, treated vessel, bifurcation lesion PCI (bifurcation with a significant side branch, as perceived by the operator, protected with a wire during PCI and/or a balloon or stent treatment of the side branch). The implantation of more than one stent in the treated vessel, stents with a diameter of less than three millimeters, glycoprotein IIb/IIIa inhibitors administration, and multivessel PCI (defined as intervention on more than one coronary artery during the initial hospitalization) were documented. Laboratory results—complete blood count, estimated glomerular filtration rate (eGFR) at admission, maximum high sensitive troponin values, duration of hospitalization, prescribed medications at discharge—were collected from the institutional patients’ database.

For follow-up, patients were contacted by phone after discharge and asked to complete a predefined questionnaire covering the following:

▪ Vital status,
▪ Ischemic events (MI, CVI, repeat PCI),
▪ Hospitalizations for cardiac causes,
▪ Adherence to antiplatelet therapy.

If a patient could not be reached, information was obtained from hospital records, treating physicians, or next of kin (with three phone attempts made before escalation). Major adverse cardio-cerebral events (MACCE: death, MI, CVI, repeat PCI) were systematically recorded.

If the patients were unavailable for follow-up, data on their status was collected from the hospital’s database, their local treating physicians, or from the next of kin. The study team member tried three times to reach the patient by phone; then, the study coordinator examined the hospital records, contacted the treating physician, and then called the next of kin listed in the hospital’s database. The study was conducted according to the declaration of Helsinki and was approved by the institutional review board.

### Statistical Analysis

Continuous data were presented as means ± standard deviation (SD). Categorical data were presented as numbers (*n*) or as percentages (%). T test and Mann–Whitney U test were used for continuous variables, while Chi-squared test and Fisher’s test were used for comparison of categorical variables. The length of follow-up in the study group was presented as the median value with interquartile range. A multivariable logistic regression model was employed to investigate the association between patient characteristics and the occurrence of major adverse cardiovascular events (MACCEs). The selection of variables for regression analysis was chosen based on known factors to influence cardiovascular outcomes: age; diabetes; left ventricular ejection fraction (LVEF); non-ST elevation myocardial infarction (NSTEMI); multivessel coronary artery disease (MVD), defined as the presence of more than 70% stenosis in two major epicardial artery territories; and decreased estimated glomerular filtration rate (eGFR). Univariate logistic regression analysis was employed as an initial step in the development of a multivariable logistic regression model to identify potential candidate variables that were individually associated with the outcome. The inclusion in the multivariable model was based on a significance threshold of *p* < 0.25.

Logistic regression was chosen due to the binary nature of the outcome variable (presence or absence of MACCE). Model selection was guided by clinical relevance and the prior literature. Adjusted odds ratios (aOR) with 95% confidence intervals (CI) were reported for each predictor. Potential multicollinearity was assessed using variance inflation factors (VIFs), and model calibration was evaluated with the Hosmer–Lemeshow goodness-of-fit test. All *p* values < 0.05 were considered statistically significant. All statistical analyses were performed using IBM SPSS Statistics 27.0 statistical software (IBM Armonk, New York, NY, USA).

## 3. Results

This retrospective, observational study included 1846 consecutive patients who were admitted to ICVD Dedinje, Belgrade, Serbia in the period 2016–2021 with a diagnosis of ACS (STEMI, NSTEMI, UA) and who were divided into two groups of patients: those older than 75 years (n = 203, 11%) and a group of patients younger than 75 years old (n = 1643, 89%). The study initially evaluated 1897 consecutive patients that suffered from ACS and fulfilled the inclusion criteria, but 27 patients were lost to follow-up, while 24 patients denied participation in the registry after being discharged from hospital and were removed from the analysis. The average age of the older than 75 years (O75) group was 80 ± 4 years, while it was 59 ± 9 years in the younger than 75 years (Y75) group. Older patients suffered more frequently from hypertension, diabetes, and peripheral arterial disease (PAD) and had previous stroke. There were a slightly more men in the younger patients group, but the difference was not significant. The patients in the O75 group had lower left ventricular ejection fraction (LVEF) (O75 36 ± 10% vs. Y75 41 ± 14%, *p* < 0.001). The clinical characteristics of the study groups are presented in Table 1. Atrial fibrillation was significantly more frequent in elderly patients (O75 30/203, 15% vs. Y75 113/1643, 7%; *p* < 0.001).

The occurrence of one of the forms of ACS (STEMI, NSTEMI, and unstable angina) was similar in both studied groups. Although older patients had significant comorbidities, a similar percentage of patients were not revascularized, i.e., received only medical therapy. Younger patients had higher values of high-sensitive (hS) troponin (Y75 49,874 ± 77,997 vs. O75 49,469 ± 63,934, *p* = 0.028), and longer hospital stays (Y75 4 ± 8 vs. O75 3 ± 3 days, *p* < 0.001), while deaths during hospitalization were more frequent in the elderly ones (O75 *n* = 9, 4.4% vs. Y75 *n* = 10, 0.6%, *p* < 0.001) (Table 2).

Older patients more frequently had multivessel coronary disease (MVD) (O75 *n* = 114, 56% vs. Y75 *n* = 727, 44%; *p* = 0.004), and the left anterior descending artery (LAD) was more often responsible for ACS in these patients (O75 *n* = 105, 52% vs. Y75 *n* = 684, 41%). In older patients, the radial approach was used less often (*n* = 148, 73% vs. *n* = 1417, 86%), probably due to the vessel size and atherosclerotic burden of the access artery. There were no differences in the interventional characteristics, except for the need to implant smaller stents in the older patients (Table 3).

**Figure 1:**
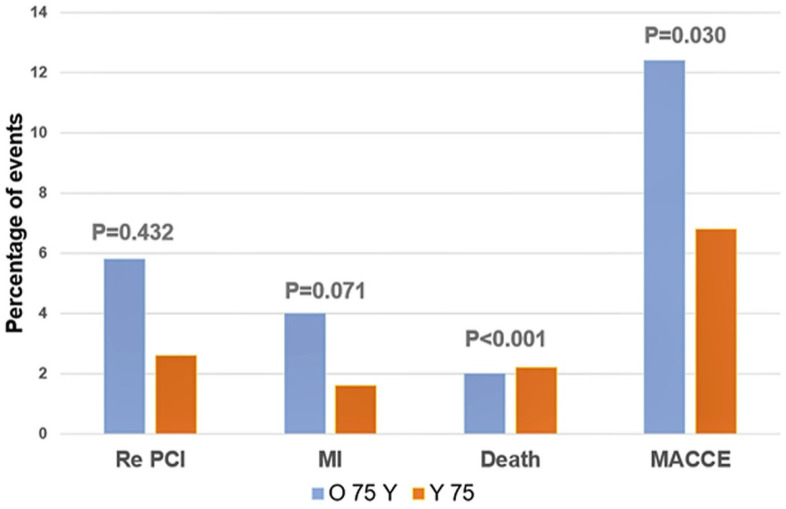
Incidence of adverse cardiovascular events.

Patients were followed for a median of 1140 days IQR [934–1325]. Major adverse events occurred more frequently in the group of patients older than 75 years, mostly due to a higher death rate (O75 *n* = 14, 6.9%, vs. Y75 *n* = 27, 1.6%; *p* < 0.001) and CVI (O75 *n* = 3, 1.5%, vs. Y75 *n* = 2, 0.1%; *p* < 0.001), while the incidence of MI and repeated interventions was similar in the both groups (Table 4, Figure 1).

**Figure 2:**
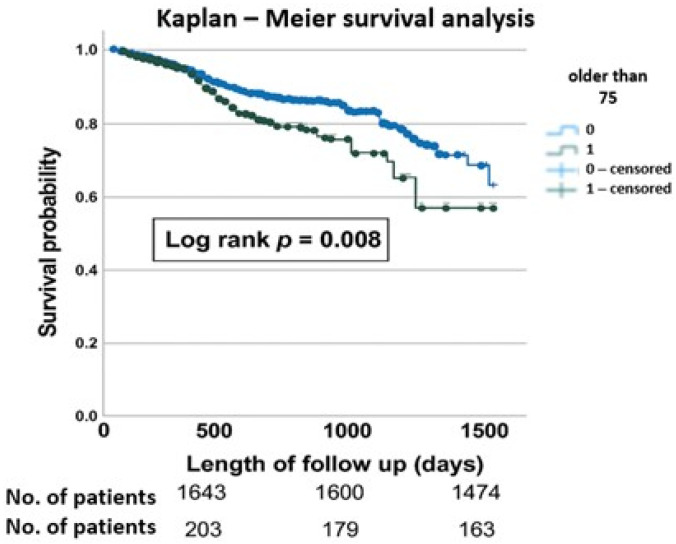
Kaplan—Meier survival analysis demonstrating difference in major adverse cardio-cerebral events (MACCE) in patients older than 75 years compared with younger patients.

Kaplan–Meier survival analysis demonstrated that elderly patients more often suffer from MACCE events, which affects their survival (log rank *p* = 0.008) (Figure 2).

In multivariable regression analysis, when compared with other significant patient characteristics like diabetes, NSTEMI, and MVD, age over 75 years and eGFR < 30 mL/min/m^2^ were not found to be independently associated with the occurrence of MACCE, the property that only MVD retained (Table 5).

Elderly patients tend to have a higher risk of bleeding. However, in our study, the occurrence of any significant bleeding according to BARC (Bleeding Association Research Consortium) class three and higher, was similar in the examined groups (Y75, 123/1643 (7.5%) vs. O75, 13/203 (6.5%), *p* = 0.587). On the other hand, the older patients were more often treated with clopidogrel as part of DAPT, which may explain the lower incidence of bleeding during the follow-up period (O75, 136/203 (67%) vs. Y75, 1025/1643 (62%); *p* = 0.043). Regarding dual antiplatelet therapy (DAPT), most of the patients were treated with a combination of aspirin and clopidogrel (1370/1846, 74.2%), while a smaller proportion of them were treated with ticagrelor (476/1846, 25.8%). Bleeding was more frequent in patients treated with ticagrelor 57/476 (12.0%) compared with clopidogrel 85/1370 (6.2%) (*p* < 0.001), mostly due to the difference in types 1 and 2 BARC bleeding: BARC 1 ticagrelor 49/476 (10.3%) vs. clopidogrel 75/1370 (5.9%), *p* < 0.001; BARC 2 ticagrelor 7/476 (1.5%) vs. clopidogrel 8/1370 (0.6%), *p* = 0.044; BARC 3 ticagrelor 1/476 (0.3%) vs. clopidogrel 2/1371 (0.1%), *p* = 0.742. Ticagrelor treatment was not associated with more adverse ischemic events, MACCE ticagrelor 51/476 (10.7%) vs. clopidogrel 172/1370 (12.5%), *p* = 0.104.

## 4. Discussion

In this retrospective observational study, we compared cardiovascular, procedural, and bleeding outcomes in patients older than 75 years in ACS compared with the younger patients when immediate coronary angiography was performed. There were slightly more men in the younger patients group, which is entirely reflective of the temporal trend in atherosclerosis development. Women tend to develop atherosclerosis later in life, but in older age, they tend to have even more complex forms of CAD and are equally represented [6]. This difference was not statistically significant in our cohort of patients. The reason for this might be the cut off point of 75 years because it does not depict postmenopausal women as a special group. Older patients suffered more frequently from hypertension, diabetes, PAD and had previous CVI. Prolonged duration of atherosclerosis due to hypertension and metabolic disturbance presented as diabetes lead to more pronounced atherosclerosis in this group [7]. This burden could be the potential cause for increased rates of adverse cardiovascular events. This was accompanied by lower LVEF. Also, they had more frequent MVD, and LAD was mostly culprit for ACS. Deaths during initial hospitalization were more frequent in the elderly group, which can be attributed to more comorbidities, frequently found MVD and slightly lower LVEF [8,9]. Surprisingly, these patients had lower values of hs Troponin, and shorter hospital stay compared with younger patients. This can be due to already present lesions in older patients, causing myocardial ischemia, where plaque rupture and vessel occlusion would produce a smaller magnitude of necrosis which was reflected in lower hs Troponin values. Several factors could be the explanation for this data. Hospital stay in ACS is strongly influenced by standardized in-hospital pathways—early coronarography/PCI, protocolized monitoring, and predefined discharge criteria—which are applied uniformly and can mitigate age-related differences. Second, discharge planning (including early transfer to rehabilitation or skilled nursing facilities) facilitates timely discharge in older patients despite a higher comorbidity burden. Notwithstanding, the incidence of all forms of ACS were similar in both age groups. This is relatively uncommon, since older patients more often suffer from NSTE—ACS, while younger patients more frequently suffer from STEMI [10]. It can be explained by the structure of the hospital admission in the study institution, since it is tertiary level cardiovascular hospital, where the patients must be previously triaged in lower-level center or by emergency medical service, which leads to higher percentage of STEMI to be admitted. The radial approach was used less often, probably due to fragility of older patients, more comorbidities and the size of the radials, which led the operators to choose femoral route for arterial cannulation, allowing larger size of guiding catheters [11].

In our study, we found that patients older than 75 years are at greater risk of MACCE events, mostly due to the higher death and CVI rate, while the other components of MACCE had similar incidence in both groups of patients. This is in accordance with large studies in this population, which suffers from advanced atherosclerosis and multiple additional comorbid conditions that yield a higher risk of repeated ischemic events and death [12]. In our study, older patients more often had LAD as the treated culprit, which can be associated with worse outcomes due to the large myocardial territory at risk. Despite more MVD, the elderly less frequently received complete revascularization, measured in terms of multivessel PCI. According to our study, almost half of the older patients presented with MVD, while only a third received multivessel PCI. The decision-making process in the study institution follows current guidelines, in which culprit-only revascularization is a primary option for most patients, while complete revascularization is usually accomplished during initial hospitalization in a staged procedure. The decision to avoid primary intervention is based on operator’s decision, while complex patients are usually evaluated in a heart team discussion. Incomplete revascularization in ACS could produce more adverse events, and patients’ age and/or functional status can determine the interventional approach in terms of the achievement of complete revascularization [12,13]. Elderly patients frequently undergo incomplete revascularization, which is associated with worse outcomes [14]. On the other hand, older patients were similarly treated in terms of complex PCI—left main interventions, bifurcations, and multivessel PCI—which can be related to more repeated MIs and PCIs [15]. Finally, older patients received a similar number of stents, but they were smaller in diameter, which can be associated with ischemic events and repeated interventions [15,16].

Older patients had more MACCEs, and this is mostly due to increased rate of death and CVI. Despite immediate invasive diagnostics and treatment, older patients still have a significant burden of concomitant diseases that have significant influence on the outcomes (Table 4, Figure 2).

However, in our study, age greater than 75 years was not an independent predictor of events when compared with other important, decisive factors like diabetes, LVEF, NSTEMI, eGFR < 30 mL/min/m^2^, and MVD. Only MVD, as a symbol of advanced coronary atherosclerosis, was independently associated with MACCE in the entire group (Table 5). Since in the majority of elderly patients, MVD and low LVEF can coexist, which may obscure the predictive effect of a single characteristic, we performed correlation matrix analysis, which yielded Pearson correlation coefficients for MVD − LVEF r = 0.012; MVD − O75 r = 0.077, and LVEF − O75 r = −0.075, while the value of VIF was 1.000, which does not clearly point towards multicollinearity. However, we cannot exclude associations between these patient characteristics that have a profound influence on survival and adverse events. This can be applied to low eGFR; low initial hemoglobin level, which can be embedded in older age; and the degree of atherosclerosis, measured as MVD.

The early invasive strategy that was uniformly applied to all these patients did not bring any substantial benefits to the elderly group compared with the younger patients. It was previously found that any invasive strategy is beneficial to the elderly, no matter early or late, compared with conservative treatment [17]. This strategy seems to offer the benefit of early revascularization, which is, in our opinion, essential for these patients.

In the MACCE composition, the difference between study groups comes mostly from the incidence of death and CVI. The difference in repeated PCI and new MI is not significant. Despite having more LAD and LM interventions and smaller stents implanted, the rates of repeated interventions remain the same. The reason for this might come from the strong influence of comorbidities on mortality after MI, which has been proven [18]. On the other hand, atherosclerosis has specific characteristics in the elderly, with fewer plaque high-risk features, which less frequently progress to critical lesions causing repeated ACS, together with a decrease in self-reporting of angina, which can lead to fewer repeated coronary angiographies and revascularization procedures [19,20].

Recommendations for medical therapy in ACS do not differ substantially for older patients, but the use of potent P2Y12 inhibitors can be associated with increased bleeding risk. In our study, older patients were more likely to receive clopidogrel compared with ticagrelor, which led to similar incidence of bleeding in both groups.

Potent platelet inhibition by ticagrelor or prasugrel offers more ischemic protection while producing more bleeding events [21,22]. Elderly patients were usually underrepresented in the trials that evaluated the use of DAPT in ACS. Our study excluded patients that had a bleeding event during initial hospitalization, which most likely influenced the overall rate of bleeding in our registry. Early bleeding events are strongly associated with adverse cardiovascular events during one year follow-up, especially death and repeated myocardial infarction. Bleeding events at the institution of DAPT are a signal of frailty, which is likely associated with cardiovascular morbidity and mortality in ACS patients [23].

Clopidogrel and ticagrelor are recommended for treatment of older ACS patients according to the latest guidelines [24], with an exception that clopidogrel may be the preferred P2Y12 inhibitor because of lower bleeding risk.

In the Elderly ACS 2 trial, prasugrel did not show superiority over clopidogrel if we compare the primary endpoints of mortality, MI, disabling stroke, hospitalization, or bleeding. On the other hand, the bleeding rate was higher in the prasugrel group [25,26]. The SWEDEHEART (Swedish Web-System for Enhancement and Development of Evidence-Based Care in Heart Disease Evaluated According to Recommended Therapies) study enrolled patients ≥ 80 years with MI who were discharged from hospital with DAPT (aspirin combined with either clopidogrel or ticagrelor). Older patients treated with ticagrelor had a 32% higher risk of bleeding, while the risk of MACCE remained similar [25,26]. The POPular AGE trial that enrolled older patients with NSTEMI showed that clopidogrel had a similar efficacy to ticagrelor in reducing MACCE events and had fewer bleeding events [27].

### Study Limitations

We analyzed retrospectively a group of patients presenting to a tertiary-level hospital with 24/7 interventional cardiology service and with onsite cardiac surgery, which might have influenced the choice of treatment. Its retrospective design may introduce potential sources of bias, including selection bias, information bias, and residual confounding, which could influence the interpretation of the findings, and these limitations must be acknowledged when analyzing study outcomes. The patient groups were heterogeneous, and comparison between them carried a risk of type I statistical error. The ratio of clopidogrel to ticagrelor usage might not reflect the modern-day use of P2Y12 inhibitors, since evidence regarding benefits and risks of potent platelet inhibition have accumulated lately. Additionally, some clinical variables may not have been uniformly collected or fully available in the medical records, which could further impact the robustness of the analysis. This includes previous medications and treatment for non-cardiovascular conditions as well as results of hemoglobin level at admission.

## 5. Conclusions

Patients older than 75 years have worse short- and long-term prognosis following ACS than younger patients. ACS treatment in patients older than 75 years presents a special challenge due to their heavy atherosclerotic plaque burden with anatomic complexities, frailty, and comorbidities. Special attention and a multidisciplinary personalized approach are needed to ensure the best possible outcome in these patients.

## Figures and Tables

**Table 1:** Clinical characteristics of ACS patients younger and older than 75 years.

Variable	O75 *n* = 203	Y75 *n* = 1643	OR [95%CI]	*p* Value
Age (years)	80 ± 4	59 ± 9		-----
Male *n* (%)	110 (54)	1003 (61)	0.758 [0.546–1.478]	0.256
Heredity *n* (%)	42 (21)	624 (38)	0.426 [0.299–0.607]	<0.001
Hypertension *n* (%)	162 (80)	1199 (73)	1.462 [1.020–2.094]	0.037
Diabetes mellitus *n* (%)	55 (27)	328 (20)	1.490 [1.069–2.077]	0.022
Insulin-dependent DM (%)	16 (8)	164 (10)	0.946 [0.754–1.120]	0.321
Dyslipidemia *n* (%)	88 (43)	644 (39)	1.188 [0.885–1.596]	0.254
PAD *n* (%)	19 (9)	68 (4)	2.392 [1.407–4.070]	<0.001
Smoking *n* (%)	36 (18)	793 (48)	0.514 [0.209–0.734]	<0.001
Previous MI *n* (%)	42 (21)	341 (21)	1.915 [0.787–1.189]	0.940
Previous CVI *n* (%)	20 (10)	93 (6)	1.821 [1.097–3.024]	0.019
Previous PCI *n* (%)	30 (15)	206 (12)	1.076 [0.742–1.965]	0.631
Previous CABG *n* (%)	14 (7)	57 (3)	1.045 [0.850–1.745]	0.054
BMI (kg/m^2^)	26 ± 4	28 ± 7	2.549 [1.473–3.626]	<0.001
LVEF (%)	36 ± 10	41 ± 14	4.275 [2.138–6.413]	<0.001

BMI—body mass index; CABG—coronary artery bypass grafting; CVI—cerebrovascular insult; DM—diabetes mellitus; LVEF—left ventricular ejection fraction; MI—myocardial infarction; PAD—peripheral arterial disease.

**Table 2:** Clinical presentation and treatment of ACS patients younger and older than 75 years.

Variable	O75 *n* = 1643	Y75 *n* = 203	OR [95%CI]	*p* Value
Unstable angina *n* (%)	8 (4)	81 (5)	0.792 [0.377–1.662]	0.256
STEMI *n* (%)	168 (83)	1358 (83)	1.007 [0.684–1.481]	0.256
NSTEMI *n* (%)	24 (12)	180 (11)	1.091 [0.693–1.716]	0.256
CABG during hosp. *n* (%)	2 (1)	10 (0.6)	1.626 [0.354–7.473]	0.256
No treatment *n* (%)	28 (14)	258 (16)	0.860 [0.564–1.309]	0.256
Days in hospital (days)	4 ± 8	3 ± 3	1.248 [0.692–1.805]	0.256
Maximum hs troponin (ng)	49,469 ± 63,934	49,874 ± 77,997	−454 [−11,911–11,001]	0.256
Death during hosp. *n* (%)	9 (4.4)	10 (0.6)	7.580 [3.043–18.884]	<0.001

CABG—coronary artery bypass grafting; NSTEMI—non-ST elevation myocardial infarction; PCI—percutaneous coronary intervention; STEMI—ST elevation myocardial infarction.

**Table 3:** Characteristics of coronary artery disease and intervention in ACS patients younger and older than 75 years.

Variable	O75 *n* = 1643	Y75 *n* = 203	OR [95%CI]	*p* Value
MVD *n* (%)	114 (56)	727 (44)	1.854 [1.106–3.458]	0.004
* Vessels treated				
LAD *n* (%)	105 (52)	684 (41)	1.500 [1.120–2.009]	0.006
Cx *n* (%)	71 (35)	457 (28)	1.056 [0.754–1.743]	0.099
RCA *n* (%)	97 (48)	699 (42)	1.237 [0.924–1.657]	0.155
SVG *n* (%)	7 (3)	33 (2)	1.744 [0.761–3.994]	0.184
Radial access *n* (%)	148 (73)	1417 (86)	0.429 [0.305–0.602]	< 0.001
Multivessel PCI *n* (%)	37 (18)	280 (17)	1.086 [0.743–1.586]	0.673
LM PCI *n* (%) (ng)	12 (6)	49 (3)	2.044 [1.068–3.911]	0.028
Bifurcation PCI *n* (%)	151 (12)	203 (12)	0.991 [0.636–1.545]	0.987
More than one stent *n* (%)	77 (38)	616 (37)	1.087 [0.879–1.245]	0.942
Stent diameter < 3.0 mm *n* (%)	72 (35)	448 (27)	1.426 [1.007–1.843]	0.031

* Vessels treated during initial hospitalization: Cx—circumflex, LAD—left anterior descending, LM—left main; MVD—multivessel coronary artery disease; PCI—percutaneous coronary intervention; RCA—right coronary artery; SVG—saphenous vein graft.

**Table 4:** Major adverse cardiovascular events during follow-up in ACS patients younger and older than 75 years.

Variable	O75 *n* = 1643	Y75 *n* = 203	OR [95% CI]	*p* Value
Death *n* (%)	14 (6.9)	27 (1.6)	4.436 [2.286–8.608]	<0.001
Myocardial infarction *n* (%)	11 (5.4)	54 (3.3)	0.502 [0.067–3.794]	0.071
CVI *n* (%)	3 (1.5)	2 (0.1)	1.017 [0.984–1.052]	<0.001
Repeated PCI *n* (%)	10 (2.0)	76 (4.6)	0.353 [0.047–2.634]	0.432
CABG *n* (%)	2 (1.0)	10 (0.7)	0.994 [0.988–1.000]	0.550
MACCE *n* (%)	40 (19.7)	169 (10.3)	1.711 [1.146–2.554]	0.030

CABG—coronary artery bypass grafting; CVI—cerebrovascular accident; MACCE—major adverse cardio-cerebral event.

**Table 5:** Univariate and multivariable logistic regression analyses to identify predictors of MACCE.

	Univariate		Multivariate	
	HR [95% CI]	*p* Value	HR [95% CI]	*p* Value
Age greater than 75	1.065 [1.065–2.300]	0.023	1.493 [0.984–2.265]	0.060
Diabetes	1.207 [0.857–1.700]	0.281	- - -	- - -
LVEF	0.991 [0.977–1.005]	0.220	- - -	- - -
NSTEMI	1.235 [0.800–1.904]	0.341	- - -	- - -
Multivessel disease	1.451 [1.085–1.940]	0.012	1.411 [1.052–1.893]	0.022
Chronic kidney disease (eGFR < 30 mL/min/m^2^)	1.776 [0.850–3.710]	0.126	1.521 [0.717–3.227]	0.274

CI—confidence interval; GFR—glomerular filtration rate; HR—hazard ratio; LVEF—left ventricular ejection fraction; NSTEMI—non-ST elevation myocardial infarction.

## Data Availability

The raw data supporting the conclusions of this article will be made available by the authors on request.
